# Stridor in Asian Infants: Assessment and Treatment

**DOI:** 10.5402/2012/915910

**Published:** 2012-02-19

**Authors:** Wong Birgitta Yee-Hang, Hui Theresa, Lee So-lun, Ho Wai-Kuen, Wei William Ignace

**Affiliations:** ^1^Division of Otorhinolaryngology-Head and Neck Surgery, Department of Surgery, Queen Mary Hospital, University of Hong Kong, Pokfulam, Hong Kong; ^2^Department of Anaesthesiology, Queen Mary Hospital, University of Hong Kong, Pokfulam, Hong Kong; ^3^Department of Paediatrics and Adolescent Medicine, Queen Mary Hospital, University of Hong Kong, Pokfulam, Hong Kong

## Abstract

Stridor is the main symptom of upper airway obstruction in infants. It can be congenital or acquired, acute or chronic. Pathologies can be located from the nose down to the trachea. Common causes include laryngomalacia, vocal cord palsy, subglottic stenosis, tracheal anomaly, laryngeal cleft, vascular and lymphatic malformation, laryngeal papillomas, craniofacial abnormalities and even head and neck tumours. In this paper, we will discuss our approach to infants with stridor including assessment with flexible and rigid endoscopy and treatments to various conditions in a tertiary centre. Causes of stridor in infants undergoing rigid laryngotracheobronchoscopy in Queen Mary Hospital, University of Hong Kong Medical Centre between 2005 and 2011 will be retrospectively reviewed. Treatments according to various conditions will be discussed. Successful management of these neonates requires accurate diagnosis, early intervention, and multidisciplinary care by ENT surgeons, paediatricians, and paediatric anaesthetists.

## 1. Introduction

Stridor is a high-pitched sound caused by turbulent air flowing through a narrowed airway. It can be inspiratory with obstruction at the supraglottic and glottis level, biphasic with obstruction at the subglottic level, and expiratory with obstruction in the trachea. Obstruction at the nasopharyngeal level often produces low-pitched inspiratory noise called stertor or snoring. Stridor in neonates can be congenital or acquired. Although the commonest cause is laryngomalacia, there are secondary airway lesions (SALs) and other causes such as vocal cord palsy, subglottic stenosis, tracheal anomaly, laryngeal cleft, vascular and lymphatic malformation, laryngeal papillomas, craniofacial abnormalities, and even head and neck tumours [1, 2]. Persistent stridor deserves careful assessment and proper investigation instead of just labeling it as a benign condition such as laryngomalacia.

## 2. Clinical Approach

 A proper history provides useful information on the differential diagnosis, severity, and level of obstruction [3]. Questions should include the nature of the stridor, inspiratory, biphasic, or expiratory; onset of stridor, aggravating factors, relationship with positioning, quality of cry, feeding difficulties, choking or aspiration, growth, perinatal history, and intubation history. For clinical assessment, the severity of respiratory distress should be first noted by examining the degree of insucking at the suprasternal and subcostal level, the degree of stridor, air entry, and relationship with positioning. Attention should also be paid to on any dysmorphic facial features, micrognathia, macroglossia, craniofacial abnormalities, voice quality, and cutaneous haemangiomas. Nasal patency can be checked by the cold-mirror test or passage of a suction catheter.

## 3. Upper Airway Assessment

 Endoscopy is the gold standard of diagnosis for paediatric airway problems. All patients referred to us with persistent stridor will be first examined awake with flexible laryngoscopy. Flexible laryngoscopy provides information on the dynamic function of the larynx [3]. Without the use of local anesthesia, the vocal cord mobility can be accurately assessed. Besides, the degree of laryngomalacia, severity of retroglossal obstruction caused by micrognathia or macroglossia, intranasal masses, choanal atresia, and tongue base lesions can be evaluated.

 Rigid laryngotracheobronchoscopy (LTB) under general anaesthesia is the second step in selected patients. Indications include patients with severe stridor, biphasic stridor, failed extubation, frequent aspiration and choking, desaturation during feeding, X-rays suggestive of subglottic or tracheal obstruction, atypical croup, and clinical suspicion with previous intubation. In our centre, rigid LTB is performed in the operation theatre by our special paediatric anaesthetist. We adopt the tubeless technique with the infant maintained under general anaesthesia and spontaneous ventilation. This allows a complete examination of the larynx in particular the subglottis and the trachea. The subglottis can be sized with different sizes of endotracheal tubes in cases suspected to have subglottic stenosis. The cricoarytenoid joints can be palpated in cases with vocal cord palsy. Diagnostic LTB can be safely performed at the day of surgery. Besides, surgical intervention can be performed in the same setting after diagnosis by LTB such as supraglottoplasty with CO_2_ laser for laryngomalacia, laser ablation for subglottic haemangiomas and laryngeal papillomas, laser resection and balloon dilatation for subglottic stenosis, and repair of cleft under microscope.

## 4. Local Data

 Infants with stridor undergoing rigid laryngotracheobronchoscopy (LTB) at Queen Mary Hospital, University of Hong Kong between January 2005 and May 2011 were retrospectively evaluated. There were a total of 138 infants, 89 males and 49 females. The diagnoses after LTB were shown up in Table 1. Laryngomalacia is the commonest cause. Secondary airway lesions (SALs) occurred in 15 out of 57 patients with laryngomalacia (Table 2).

## 5. Treatment

 Accurate diagnosis allows early intervention to relief upper airway obstruction and recovery of normal airway function. Majority of surgery could be safely performed in the early infantile period including laser surgery and repair, surgical resection of tumours, and reconstruction of the pharynx and skull base [4]. This could avoid prolonged intubation or tracheostomy. Other advancement in medical treatment includes the use of propranolol in subglottic haemangiomas.

### 5.1. Laryngomalacia

**Figure 1:**
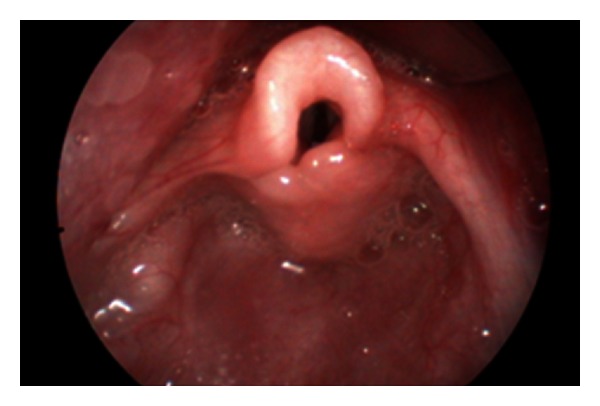
Endoscopic features of laryngomalacia.

**Figure 2:**
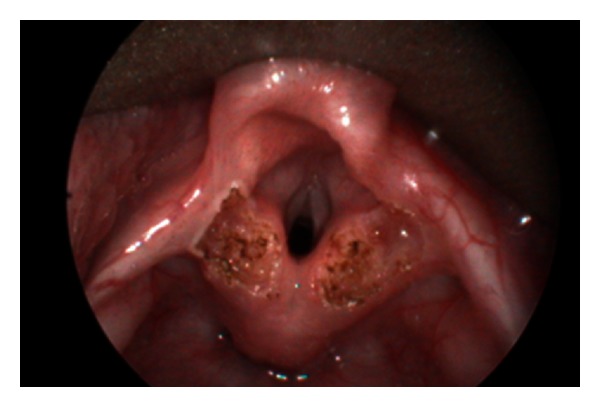
Laser aryepiglottoplasty under tubeless anaesthetic technique.

 Laryngomalacia is the commonest cause of stridor in infants. It usually presented as inspiratory stridor at the first few days or weeks of life. Although the majority of cases are self-limiting, severe stridor with respiratory distress, feeding difficulty, or failure to thrive deserves investigation and intervention. Flexible laryngoscopy demonstrates endoscopic features of laryngomalacia with tubular epiglottis, short aryepiglottic fold, and redundant arytenoids mucosa. Rigid laryngotracheobronchoscopy is useful to further rule out secondary airway lesions (SALs) which occurred in 26% of laryngomalacia in our series. The prevalence varies from 7.5% to 64% in the literature [5]. The commonest SAL in our series is subglottic stenosis followed by tracheomalacia which is compatible to the literature [5]. As a tertiary referral centre, most of the cases referred to us have severe laryngomalacia with feeding difficulty, choking, or failure to thrive requiring intervention. Laser aryepiglottoplasty with CO_2_ laser can be safely performed under spontaneous ventilation and tubeless technique to resect the redundant arytenoids mucosa and to release the short aryepiglottic folds (Figure 1 and Figure 2). Postoperatively, our patient will be given steroid and monitored in intensive care unit for one day. Feeding can be resumed on the day of the surgery and patients can be discharged on the second day after operation.

### 5.2. Vocal Cord Palsy

 Bilateral vocal cord palsy often presented as stridor shortly after birth. The majority are idiopathic while others are secondary to pathologies of the central nervous system such as Arnold-Chiari malformation, hydrocephalus, or cerebral palsy [1]. Cases with respiratory distress require tracheostomy. Spontaneous recovery may occur in some patients, while definitive surgery for decannulation such as vocal cord lateralization, cordectomy, or arytenoidectomy could be performed in adolescence period. Unilateral vocal cord palsies are usually iatrogenic cause after cardiac surgery or PDA ligation. Fortunately, most of them are well compensated and well tolerated. In our centre, patients with glottic insufficiency or aspiration can undergo injection laryngoplasty with hyaluronic acid or fat to medialise the palsied vocal cord.

### 5.3. Subglottic Stenosis

**Figure 3:**
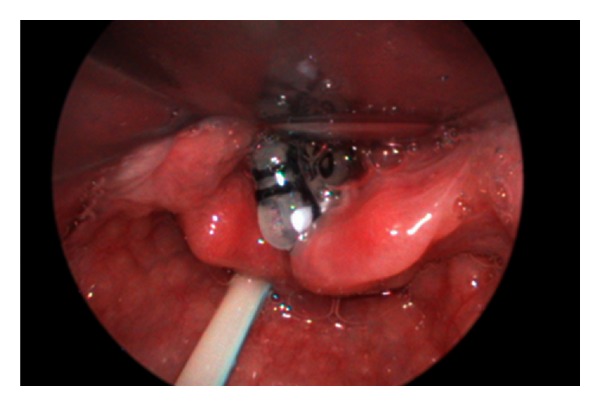
Endotracheal tube with superior end sutured as laryngeal stent after laryngotracheal reconstruction.

 Subglottic stenosis can be congenital or acquired. It is defined as a diameter of less than 4 mm of the cricoid region in a full-term infant and less than 3 mm in a premature infant [1, 2]. Accurate assessment and sizing of the subglottis can be performed during rigid laryngotracheobronchoscopy with different sizes of endotracheal tubes. Length of the stenotic segment can also be measured. In our centre, Cotton-Myer grade I and II subglottic stenosis with excess fibrous connective tissue or granulations can be treated with multiple sessions of CO_2_ laser under spontaneous ventilation and tubeless technique to avoid a tracheotomy. Severe subglottic stenosis will necessitate tracheostomy followed by laryngotracheal reconstruction (LTR) with rib cartilage to augment the subglottis. An age-appropriate size endotracheal tube specially trimmed with the upper end sutured to prevent aspiration was placed as a laryngeal stent for 6 weeks until the rib graft was taken (Figure 3).

### 5.4. Subglottic Haemangioma

 Subglottic haemangioma is usually presented by 6 months of age as inspiratory or biphasic stridor. 50% of these infants have concomitant cutaneous haemangiomas. Unilateral subglottic haemangiomas can be successfully treated with CO_2_ laser ablation and systemic steroid. In the past, the majority of circumferential haemangiomas required tracheostomy although open submucosal resection has been performed. In 2008, a paper on the first successful use of oral propranolol for cutaneous haemangiomas in 11 children was published [6]. This was followed by 2 papers of propranolol therapy on subglottic haemangiomas [7, 8]. The mechanism of action is that propranolol induces apoptosis and decreases the production of endothelial vascular and fibroblastic growth factors (VEGFs and FGF*β*). One of our patients, a 6-month-old girl, presented with extensive haemangioma involving the subglottis, posterior pharyngeal wall, and cervical esophagus. Tracheostomy was performed for the circumferential haemangioma. However, there was no sign of resolution or shrinkage of the haemangioma at the age of two. She was then put on propranolol therapy. The haemangioma was completely resolved after 6 months of treatment.

### 5.5. Laryngeal Cleft

**Figure 4:**
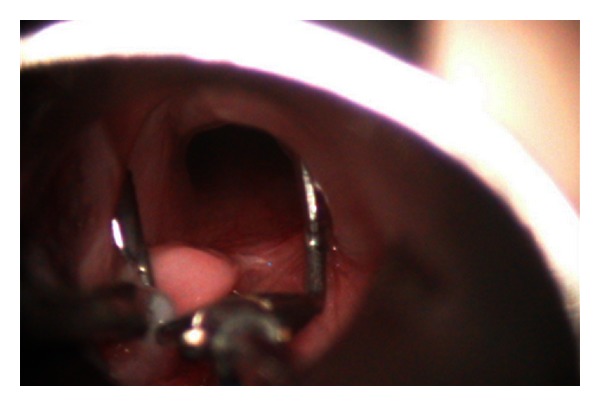
Laryngeal cleft demonstrated with the use of vocal cord spreader.

 Laryngeal cleft is a rare condition. The patient presented as stridor, aspiration, and recurrent pneumonia [9]. Diagnosis can be missed with flexible laryngoscopy as the larynx may appear as normal. In suspected cases, the posterior arytenoids should be examined and palpated during rigid laryngotracheobronchoscopy. The extent can be nicely shown with a vocal cord spreader (Figure 4). Patients with type I and II laryngeal cleft can try medical and feeding therapy [10]. However, if aspiration or pneumonia is frequent, repair can be performed endoscopically [10, 11]. In our practice, we advocate the use of spontaneous ventilation and the tubeless anaesthetic technique. This allows an adequate and excellent exposure for repair. A V-shaped incision was made around the cleft to create a raw surface. The mucosa was then approximated with 5/0 vicryl, and an age-appropriate endotracheal tube was inserted as a laryngeal stent to allow healing of the wound. The patient was then transferred to intensive care unit for 10 days and extubated when the wound was healed.

### 5.6. Congenital Head and Neck Tumours

 Congenital head and neck tumours can lead to stridor and upper airway obstruction in newborns. Differential diagnoses include teratomas, haemangiomas, lymphatic malformations, and neuroglial heterotopias [12, 13]. Nowadays, these tumours can be detected early in the antenatal period by fetal ultrasound or MRI. Well-planned delivery by EXIT (ex-utero intrapartum treatment) has been carried out in our patient with anticipated airway obstruction caused by a huge neuroglial heterotopias [4]. This is an extension of standard Caesarean section with the baby partially delivered while the umbilical cord was kept intact to maintain maternal-fetal circulation to allow time for intubation and to secure the airway. In our centre, we advocate early resection of head and neck tumours in the infantile period [4]. Pharyngeal defects after resection in infants can be safely reconstructed with a locoregional flap created by the excessive skin, while the skull base defect can be reconstructed by spitting the cranial bone [4]. Early resection and reconstruction can avoid prolonged intubation or tracheostomy and thus allow early recovery of normal airway function.

## 6. Conclusion

 Stridor in infants can be due to different causes other than laryngomalacia. Assessment should start from careful history taking and clinical examination. Persistent symptoms deserve referral to otorhinolaryngologists for flexible and rigid endoscopy. Surgical intervention from laser surgery to major resection and reconstruction could be safely performed in early infantile period.

## Figures and Tables

**Table 1:** Diagnosis after laryngotracheobronchoscopy (LTB).

Diagnosis	No. of patients (*N* = 138)
Laryngomalacia	57
Vocal cord palsy	8
Subglottic stenosis	20
Tracheal stenosis	5
Tracheomalacia	4
Vallecula cyst	5
Subglottic haemangioma	3
Laryngeal cleft	2
Craniofacial abnormality	15
Respiratory papillomatosis	4
Choanal atresia	3
Vocal cord nodules	8
Head and neck tumours	4

**Table 2:** Synchronous airway lesions (SALs) associated with laryngomalacia.

Synchronous airway lesion (SAL)	No. of patients (*N* = 15)
Subglottic stenosis	5
Vallecula cyst	4
Tracheomalacia	4
Vocal cord palsy	2
